# Cytomegalovirus-specific T-cells are associated with immune senescence, but not with systemic inflammation, in people living with HIV

**DOI:** 10.1038/s41598-018-21347-4

**Published:** 2018-02-28

**Authors:** Vibe Ballegaard, Peter Brændstrup, Karin Kaereby Pedersen, Nikolai Kirkby, Anette Stryhn, Lars P. Ryder, Jan Gerstoft, Susanne Dam Nielsen

**Affiliations:** 1 Viro-immunology Research Unit, Department of Infectious Diseases, Rigshospitalet, University Hospital of Copenhagen, Copenhagen, Denmark; 2 Department of Clinical Immunology, Rigshospitalet, University Hospital of Copenhagen, Copenhagen, Denmark; 3 Department of Immunology and Microbiology, University of Copenhagen, Copenhagen, Denmark; 4 Department of Hematology, Herlev University Hospital, Herlev, Denmark; 5 Department of Medical Microbiology, Rigshospitalet, University Hospital of Copenhagen, Copenhagen, Denmark

## Abstract

In people living with HIV (PLWHIV), coinfection with cytomegalovirus (CMV) has been associated with inflammation, immunological ageing, and increased risk of severe non-AIDS related comorbidity. The effect of CMV-specific immune responses on systemic inflammation, immune activation and T-cell senescence was evaluated in 53 PLWHIV treated with combination antiretroviral therapy (cART). Activated-, terminally differentiated-, naïve-, and senescent T-cells were assessed by flow cytometry, and plasma levels of CMV IgG, interleukin-6, tumor necrosis factor-α, high-sensitivity C-reactive protein and soluble-CD14 were measured. In PLWHIV, expression of interleukin-2, tumor necrosis factor-α and interferon-γ was measured by intracellular-cytokine-staining after stimulation of T-cells with CMV-pp65, CMV-IE1, and CMV-gB. Increased CMV-specific T-cell responses were associated with a higher ratio of terminally differentiated/naïve CD8+ T-cells and with increased proportions of senescent CD8+ T-cells, but not with systemic inflammation or sCD14. Increased CMV-specific CD4+ T-cell responses were associated with increased proportions of activated CD8+ T-cells. In PLWHIV with expansion of CMV-specific T-cells or increased T-cell senescence, CMV-specific polyfunctionality was maintained. That the magnitude of the CMV-specific T-cell response was associated with a senescent immune phenotype, suggests that a dysregulated immune response against CMV may contribute to the immunological ageing often described in PLWHIV despite stable cART.

## Introduction

After introduction of combination antiretroviral therapy (cART), life expectancy has increased for people living with HIV (PLWHIV)^1–3^, but has not yet reached that of the background population^4^. Non-AIDS comorbidity contributes to the gap in life expectancy, and PLWHIV on stable cART have increased risk for early onset of age-related diseases including cardiovascular diseases and renal diseases^5^. This is probably due to complex interactions between HIV infection itself, traditional risk factors, and other factors such as coinfection with cytomegalovirus (CMV), residual immune dysfunction, and inflammation^6,7^.

The majority of PLWHIV are coinfected with CMV, a common β-herpes virus that establishes lifelong latent infection with frequent asymptomatic reactivations^8^. In PLWHIV, the presence of CMV coinfection has been associated with increased risk of inflammation, phenotypic T-cell alterations, and non-AIDS comorbidities^9–15^. CMV seropositivity in PLWHIV have been associated with expansion of CD8+ T-cells, a reduced CD4+/CD8+ T-cell ratio, and increased levels of CD8+ T-cell senescence markers^9,10,12,14,16^. Characteristics that independently have been associated with increased morbidity and mortality^17–19^. The immunological mechanisms are incompletely understood, and it has been suggested that not only CMV infection itself but also the host’s immune response against CMV could drive these changes. In treated HIV infection, the magnitude of the CMV-specific immune response, defined by CMV IgG levels or CMV-specific T-cell responses, has been associated with phenotypic T-cell alterations^15,20–23^, and non-AIDS comorbidity^24–29^, suggesting that a dysfunctional control of CMV may contribute to the immune dysfunction and early onset of age-related comorbidity observed in PLWHIV despite treatment with cART. However, in many of the previous studies confounders could significantly affect the conclusions, and to our knowledge the relationship between CMV-specific immune responses and inflammation or phenotypic T-cell alterations have not previously been evaluated in a well-treated low-morbidity cohort of PLWHIV. In addition, most previous studies used CMV IgG as a marker of CMV burden, and few studies have investigated the impact of the CMV-specific T-cell function on those associations.

In previous studies we found that PLWHIV had increased immune activation, inflammation, and microbial translocation compared to matched controls^30–32^. In the cohort of the present study, CMV coinfection was detected in 92% of PLWHIV, and we hypothesized that increased CMV IgG levels and total CMV-specific T-cell responses against CMV-pp65, CMV-IEI, and CMV-gB, would be associated with increased inflammation, immune activation, and T-cell senescence in PLWHIV. We further evaluated whether PLWHIV maintain CMV-specific T-cell polyfunctionality, defined as single cells producing two or more cytokines, despite increased T-cell senescence and higher CMV-specific T-cell responses.

## Methods

### Study population

Sixty-one PLWHIV were recruited from the outpatient clinic at the Department of Infectious Diseases, University Hospital of Copenhagen, Rigshospitalet, in a study regarding cardiovascular risk profile and cognitive function with measurements of physical, immunological, inflammatory, and cognitive parameters. Results from the study have previously been published in detail^30–33^. For comparison, 31 healthy individuals matched for age, gender, education and comorbidity were included. Nineteen of the controls also participated in a study on diabetes^34^.

CMV coinfection (defined as serum CMV IgG >5 U/mL) was detected in 92% (n = 56) of PLWHIV and 64% (n = 18) of the controls. CMV-seronegative individuals or individuals without available serum samples were excluded from the present study. All participants had received cART for a minimum of 2 years prior to inclusion (median duration of treatment 7.6 years) and had suppressed viral replication <500 copies/mL for at least 1 year before inclusion. Median CD4+ T-cell count was 540 cells/µL. Exclusion criteria were acute illness, chronic infection with hepatitis B virus (HBV) or hepatitis C virus (HCV), intravenous drug use, autoimmune disease, cancer, or pregnancy.

The study was approved by the National Committee on Biomedical Research Ethics for the Capital Region of Denmark (H-2-2010-089) and the Danish Data Protection Agency and conducted in accordance with the second declaration of Helsinki. Written informed consent was obtained from all participants.

Fasting venous blood samples were collected. Routine evaluation included CD4+ T-cell count (cells/uL) and HIV viral load (HIV-RNA, copies/ml). Nadir CD4+ T-cell count was recorded as the lowest CD4+ T-cell count in each individual’s history. Interleukin-6 (IL-6), tumor necrosis factor-alpha (TNF-α), high-sensitivity C-reactive protein (hsCRP) and soluble-CD14 (sCD14), were measured in heparinized plasma as previously described^30–32^. T-cell subsets, defined as percentage of activated (CD38+ HLA-DR+), naive (CD45RA+ CD27+ CCR7+), terminal differentiated (CD45RA+ CD27-CCR7-) and senescent (CD28-CD57+) cells among CD4+ and CD8+ T-cells, were measured immediately after sampling of peripheral blood collected in EDTA tubes, incubated with fluorescent dye–conjugated monoclonal antibodies, and analyzed with six-colour FACSCanto flow cytometer (Becton Dickinson, Franklin Lakes, NJ) as previously described^30^.

### CMV IgG antibody levels and avidity

CMV IgG antibody levels were measured in frozen serum samples from all participants using a commercial fully automated chemiluminescent immunoassay (LIAISON ® CMV IgG II, DiaSorin S.P.A., Saluggia, Italy), and according to the manufacturer’s instructions^35^. Samples with CMV IgG levels >180 U/mL were reanalysed in 1:5 dilutions. Plasma CMV DNAemia was assessed using the Amplicor CMV Monitor test (Roche Diagnostics, Indianapolis, IN). To differentiate between primary and chronic CMV infection, the antigen-binding avidity of CMV IgG antibodies in serum was measured with the LIAISON ® CMV Avidity II assay (DiaSorin S.P.A., Saluggia, Italy)^36^. The signal of an untreated sample was compared with the signal of the same sample after treating with urea (avidity index). An avidity index of ≤0.2 suggests acquisition of primary infection less than 3 months prior to sample collection^37^.

### Cell preparation and peptide pool stimulation

Stimulation of peripheral blood mononuclear cells (PBMC) with peptide-pools from CMV-pp65, CMV-IE1, and CMV-gB followed by intracellular-cytokine-staining, was only performed in PLWHIV and not in HIV-uninfected controls. PBMC were isolated from whole blood by density gradient centrifugation, re-suspended in supplemented RPMI-media containing 10% dimethylsulfoxid (DMSO) and cryopreserved in liquid nitrogen. Before stimulation, frozen PBMC were thawed quickly in 37 °C, washed twice with complete RPMI, and rested overnight in complete RPMI containing 10% heat-inactivated foetal bovine serum (density 2.5 × 10^6^ cells/mL). PBMC were stimulated in round-bottom tubes in duplicate samples at a density of 1 million/300 µL of complete RPMI medium using either a CMV-pp65 peptide pool containing 138 peptides derived from a peptide scan (15mers with 11 aa overlap) through 65 kDa phosphoprotein (pp65) (Swiss-Prot ID: P06725), a CMV-IE-1 peptide pool of 120 peptides derived from a peptide scan (15mers with 11 aa overlap) through 55 kDa immediate-early protein 1 (IE-1) (Swiss-Prot ID: P13202), or a CMV-gB peptide pool of 224 peptides derived from a peptide scan (15mers with 11 aa overlap) through Envelope glycoprotein B (gB) (Swiss-Prot ID: P06473). Staphylococcal enterotoxin B (SEB) (2.5 ug/mL; Sigma-Aldrich) dissolved in DMSO (1 µg/mL) was used as a positive control. Cells were incubated with CMV-pp65, CMV-IE1, CMV-gB peptide pools (1 µg/mL per peptide, PepMix, JPT Peptide Technologies) or SEB in the presence of co-stimulatory anti-CD28/CD49d (1 µg/mL, BD Biosciences) for six hours at 37 °C^38,39^. Brefeldin A (1 µg/mL, BD Biosciences) was added after 2 hours. An unstimulated control was incubated with DMSO, anti-CD28/CD49d, and Brefeldin A in order to detect background staining.

### Intracellular cytokine assays and polyfunctionality analyses

At the end of stimulation, cells were incubated with ethylene-diamin-tetra-acetate (EDTA)-solution, washed with fluorescence-activated cell sorting (FACS) washing buffer, stained with BD Horizon™ Fixable Viability Stain 450 (FVS450), and subsequently treated with FACS lysing- and FACS permeabilization solution (BD FACS™) and stained with anti-CD4-FITC/anti-CD69-PE/anti-CD3-PerCP (clone SK3/L78/SK7 BD Fastimmune™), anti-CD8-V500 (clone SK1 BD Horizon™), anti-IL-2-BV421 (clone 5344.111 BD Horizon™), anti-TNF-α-APC (clone 6401.1111 BD FastImmune™) and anti-IFN-γ-PE-Cy7 (clone B27 BD Pharmingen™), at RT for 30 minutes. After washing, cytokine responses were acquired immediately using a BD FACSCanto™ II flow cytometer.

Flow cytometry results were analysed using BD FACSDiva (v8.0.1) software (BD Biosciences). A lymphocyte gate based on FSC/SSC, a singlet gate, and a live/dead cell gate were applied before gating on CD3+ CD4+ and CD3+ CD8+ cells. Further, for each T-cell subset, CD69+ populations were gated from CD69+ histograms for CD4+ and CD8+ populations, and expression of IFN-γ, TNF-α and IL-2 was then determined from the CD4+ and CD8+ populations (Supplementary Figure 1). To obtain co-expression patterns a combinational gating strategy was applied to obtain all functional subsets. Net subset frequencies were determined by background subtraction (with co-stimulation but no peptide antigen). A positive response was defined as a background-subtracted response above 1/10.000 of CD4+ or CD8+ (>0.01% of CD8+ or CD4+) and at least 40 events. In each participant at least 100.000 events were recorded. CMV-IE1-specific T-cell responses were below the cut-off in 26% of the polyfunctional CD8+ T-cell subsets and in 77% of the polyfunctional CD4+ T-cell subsets in PLWHIV, and were excluded from further statistical analysis. By summing up the frequency of CD4+ or CD8+ T-cells within each unique combination of functions (IFN-γ, TNF-α, or IL-2), we analysed the magnitude of the total CMV-specific response (%CD8+ or %CD4+). Thus, each responding cell was calculated only once. Polyfunctionality visualization and analysis was performed using Spice Version 4.2.3 (Mario Roederer, ImmunoTechnology Section, VRC/NIAID/NIH) software^40^.

### Statistical analysis

**Figure 1:**
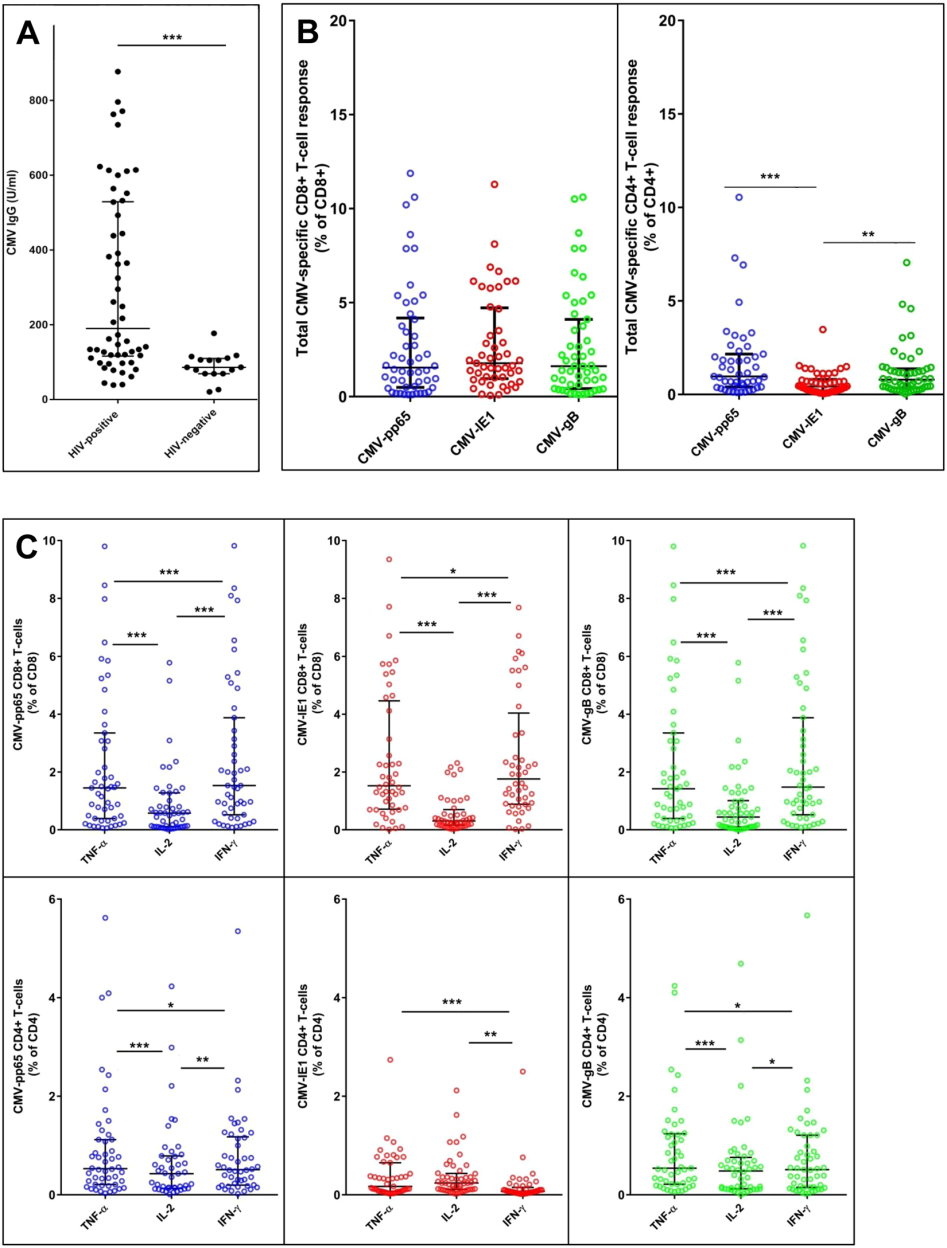
CMV-specific immune responses in PLWHIV. In a cohort of PLWHIV, CMV IgG antibody levels were measured, and CMV-specific CD8+ and CD4+ T-cell responses were examined by measuring intracellular expression of IFN-γ, TNF-α, and IL-2 after stimulation with CMV-pp65, CMV-IE1, and CMV-gB. By summing up the frequency of CD4+ or CD8+ T-cells within each unique combination of functions (IFN-γ, TNF-α or IL-2), the magnitude of the total CMV-specific response (%CD8+ or %CD4+) was analysed. (**A**) CMV IgG antibody levels in PLWHIV and HIV-negative age-matched controls (190 U/mL (118–528) versus 86 U/mL (69–109). (**B**) Total CMV-pp65-, CMV-IE1-, and CMV-gB-specific CD8+ and CD4+ T-cell responses. (**C**) Frequency of CMV-specific CD8+ and CD4+ T-cells expressing IFN-γ, TNF-α, and IL-2. In (**B**) and (**C**), blue circles represent CMV-pp65, red circles represent CMV-IE1, and green circles represent CMV-gB. For each variable, median and interquartile range are shown. Mann-Whitney test was performed to compare PLWHIV with uninfected controls in (A). Wilcoxon paired signed-rank test was used to compare frequency of CMV-specific CD8+ and CD4+ T-cells in (**B**) and (**C**). *p < 0.01, **p < 0.001, ***p < 0.0001. Bonferroni corrected significance level for (**B**) and (**C**) with 3 end points evaluated; p < 0.017.

Data are given as mean and standard deviation for normally distributed continuous data or as median and interquartile range for skewed data. Categorical data are given as percentage and total number. CMV IgG levels in age-matched CMV-seropositive HIV-negative individuals (median 86 U/mL, interquartile range (IQR) 69–109) were used as a control group, and the 97.5th percentile (177 U/mL) were chosen as a cut-off defining a threshold between a low and a high CMV IgG response (Fig. 1A). PLWHIV were separated in two groups according to this cut-off, defining a normal and a high CMV IgG response group, respectively. CMV IgG response groups were compared with Student t-test or Mann Whitney test for continuous variables and with Χ^2^ test for categorical variables. Continuous data were analysed for variance with log-transformation of skewed data to obtain normal distribution. Differences in cytokine responses among CD4+ and CD8+ T-cells were evaluated by the nonparametric Wilcoxon signed-rank test for paired samples and Mann-Whitney U test for independent samples.

Univariate and multivariate linear regression models with immune activation, immune senescence and systemic inflammation as dependent variables and CMV-specific immune responses as independent variables were created. All variables were log2-transformed in order to meet assumptions for linear regression. Using a backward selection approach on variables with known or potential impact on immune activation, senescence or inflammation in PLWHIV (age, gender, nadir CD4+ T-cell count, current CD4+ T-cell count, years since diagnosis, pre-ART viral load, smoking and BMI) models were minimized to include only age and gender as covariates, and separate models with additional inclusion of a single HIV-associated variable to each model were created. Bonferroni correction and false discovery rate (Benjamini-Hochberg method) adjusted p-values were applied to adjust for multiple testing when appropiate. Statistical analysis was performed using SAS (version 9.4 SAS Institute, Copenhagen, Denmark), graphic presentation with GraphPad Prism 7 (GraphPad Software Inc), and SPICE 5.35 (Mario Roederer, Immuno Technology Section, VRC/NIAID/NIH) software was used to analyse polyfunctionality^40^.

### Data availability

The datasets generated during and/or analyzed during the current study are available from the corresponding author on reasonable request.

### Take-home message

CMV-specific T-cell responses are independently associated with markers of immune senescence after adjustment for HIV-related factors.

## Results

### CMV IgG levels and CMV-specific T-cells in PLWHIV on cART

PLWHIV had higher CMV IgG levels than matched HIV-negative individuals (190 U/mL (118;528) versus 86 U/mL (69;109), p < 0.0001) (Fig. 1A). Current CMV reactivation or primary CMV infection was not detected, since none of the participants had detectable CMV DNA in plasma, and all had an avidity index above 0.20. PLWHIV had lower levels of CD4+ T-cells and higher levels of CD8+ T-cells, resulting in a decreased CD4+/CD8+ T-cell ratio (p = 0.0007), when compared to matched HIV-negative individuals (Table 1). Furthermore, levels of IL-6 and TNF-α were higher in PLWHIV than in HIV-negative individuals (Table 1). CMV-specific CD4+ and CD8+ T-cell cytokine responses against the immunodominant CMV-antigens CMV-pp65, CMV-IE1, and CMV-gB were only measured in PLWHIV. Representative CD8+ and CD4+ T-cell responses and flow cytometry gating strategy from one HIV-positive individual is presented in Supplementary Figure 1. After background subtraction, a positive CMV-specific T-cell response was detected in 94% of PLWHIV. Three CMV-seropositive individuals were non-responders in all assessed T-cell functions.

**Table 1:** Comparison of CMV seropositive PLWHIV and HIV-uninfected controls.

CMV IgG, U/mL	*PLWHIV (n* = *54)*	*Controls (n* = *16)*	*PLWHIV divided according to CMV IgG*	
	190 (118–528)	86 (69–109)***	*CMV IgG* < 177 *(n* = *27)*	*CMV IgG* ≥ *177* *(n* = *27)*
**Clinical characteristics**				
Age, years	*50* ± *7*	*52* ± *6*	50 ± 8	51 ± 7
Gender, male	*89 (48)*	*75 (12)*	93 (27)	85 (23)
Years since diagnosis	*9.0 (6.0–12.0)*	*NA*	8 (5.0–12.5)	10 (6.0–12.0)
CD4+ nadir, cells/µL	*160 (62–280)*	*NA*	210 (120–350)	110 (58–260)
CD4+, cells/µL	***592*** ± ***256***	***906*** ± 177**	621 ± 258	562 ± 255
CD8+, cells/µL	***747*** ± ***295***	***554*** ± 136*	692 ± 273	804 ± 313
**Markers of inflammation**				
IL-6, pg/mL	***2.2 (1.2–3.0)***	1.26 (1.0–1.6)*	2.2 (1.1–3.3)	2.0 (1.2–2.9)
TNF-α, pg/mL	***2.7 (2.3–3.2)***	2.2 (1.9–2.4)**	2.6 (2.3–3.2)	2.8 (2.4–3.5)
hs-CRP, ug/mL	*1.1 (0.6–2.4)*	*0.9 (0.5–2.8)*	1.1 (0.7–1.6)	1.1 (0.5–2.6)
**Markers of immune activation**				
CA CD8+, %	*5.8 (3.0–7.5)*	*NA*	5.9 (3.0–8.0)	5.6 (3.0–7.3)
CA CD4+, %	*1.6 (1.0–2.1)*	*NA*	1.5 (0.9–2.0)	1.7 (1.0–2.4)
sCD14, pg/mL	*891 (381–1903)*	*NA*	941 (399–1903)	815 (381–1648)
**Markers of immune senescence**				
CD4+/CD8+ ratio	**0.8 (0.6–1.1)**	1.7 (1.5–2.0)***	**0.9 (0.7–1.2)**	**0.6 (0.5–0.9)** *
TD/N CD8+	1.6 (0.7–3.2)	NA	1.3 (0.6–2.6)	2.2 (0.8–4.3)
TD/N CD4+	0.07 (0.01–0.17)	NA	0.07 (0.01–0.14)	0.06 (0.01–0.19)
Senescent CD8+, %	*28.9 (17.2–36.3)*	*NA*	29.6 (14.7–37.3)	28.3 (17.2–35.6)

Data are presented as medians and interquartile range (IQR) for skewed data, mean and standard deviation (±SD) for normally distributed data, and categorical data as percentage (%) and number (n). Clinical characteristics marked in italic has previously been presented^30–32^. CMV IgG levels in HIV-uninfected individuals were used as a reference group with the 97.5th percentile (177 U/mL) defining the cut-off between PLWHIV with low and high CMV IgG levels. Differences between groups were tested with Student *t*-test, Mann Whitney test or *Χ*^2^ test. T-cell subsets were defined as follows: Activated (CA) (CD38+ HLA-DR+), terminally differentiated (TD) (CD45RA+ CD27-CCR7-), naïve (N) (CD45RA+ CD27+ CCR7+), and senescent (CD28-CD57+) T-cells. Flow cytometry results are given as percentage of CD4+ or CD8+ T-cells. P-values in bold for P < 0.05. ***P < 0.001, ** P < 0.005, *P < 0.05.

Total CMV-specific CD8+ T-cell responses were of the same magnitude for all three epitopes (CMV-pp65; 1.65 (0.53;4.18), CMV-IE1; 1.72 (0.93;4.38), CMV-gB; 1.62 (0.43;4.11), p = 0.810, Fig. 1B). In CD4+ T-cells, the magnitude of the total CMV-pp65- and CMV-gB-specific responses were significantly higher than the total CMV-IE1-specific responses (CMV-pp65; 0.97(0.40;2.16), CMV-IE1; 0.43 (0.18;0.81), CMV-gB; 0.79 (0.35;1.37), CMV-pp65 vs. CMV-IE1; p < 0.0001, CMV-gB vs. CMV-IE1; p < 0.005, Fig. 1B). In addition, total CMV-specific responses were higher in CD8+ T-cells than in CD4+ T-cells (CMV-pp65; p = 0.006, CMV-IE1; p < 0.0001, CMV-gB; p < 0.0001). CMV-pp65-, CMV-IE1-, and CMV-gB-specific IFN-γ-, TNF-α-, and IL-2- T-cell responses and profiles of polyfunctionality are depicted in Fig. 1C and Supplementary Figure 2, respectively.

**Figure 2:**
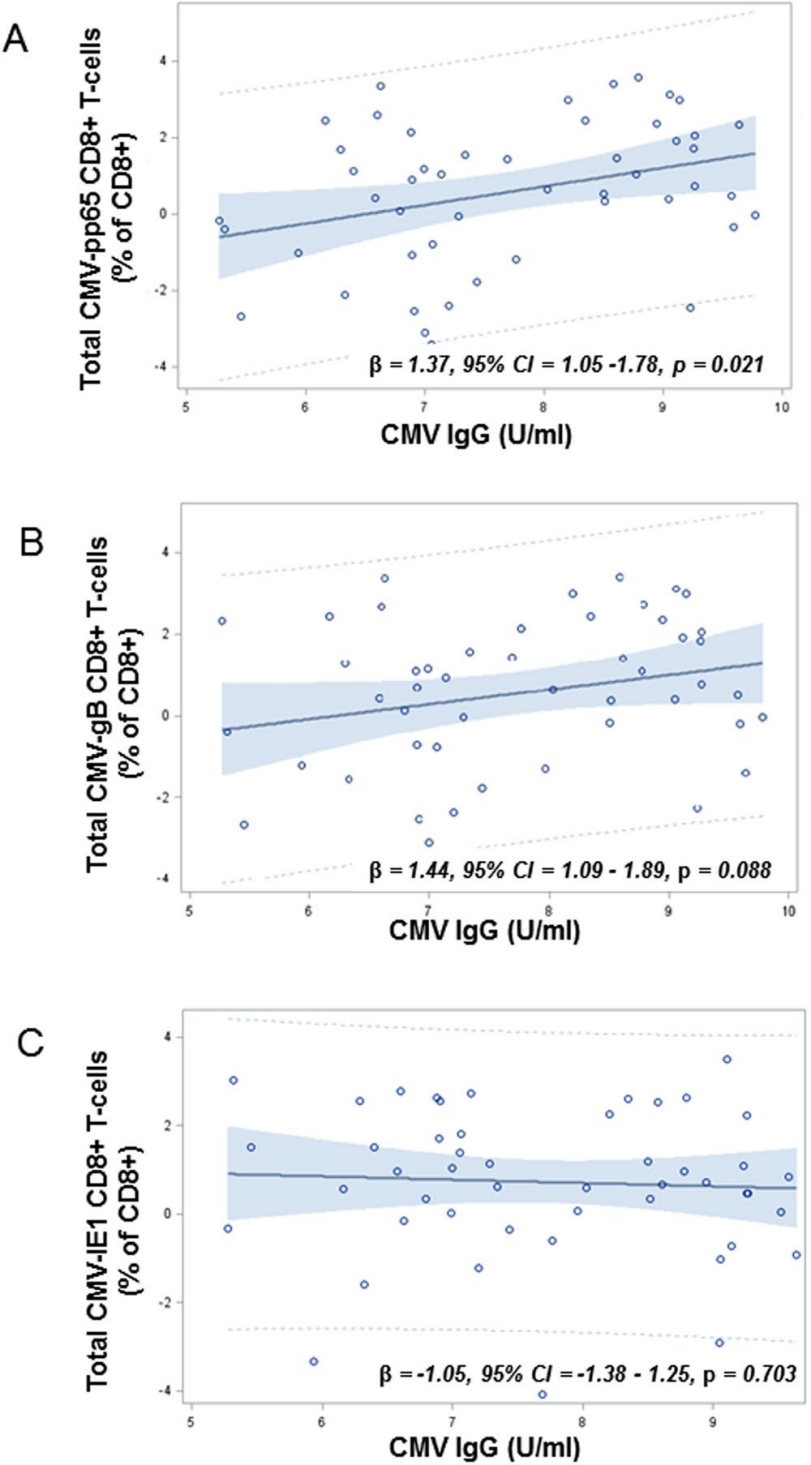
Relationship between CMV IgG levels and CMV-specific T-cell responses in PLWHIV. Associations between total CMV-specific T-cell responses and CMV IgG levels for total CMV-pp65-specific CD8+ T-cell responses (**A**), total CMV-gB-specific CD8+ T-cell responses (**B**), and total CMV-IE1-specific CD8+ T-cell responses were characterized.

The relationship between CMV IgG levels and total CMV-specific T-cell responses in PLWHIV were investigated in univariate and multivariate regression models. CMV IgG levels were associated with total CMV-pp65 CD8+ T-cell responses (β = 1.37, 95% CI = 1.05;1.78, p = 0.021), but not with total CMV-gB-specific CD8+ T-cell responses (β = 1.44, 95% CI = 1.09;1.89, p = 0.088) or total CMV-IE1-specific CD8+ T-cell responses (β = −1.05, 95% CI = −1.38;1.25, p = 0.703) (Fig. 2). Adjustment for age, gender and nadir CD4+ did not alter the results. No association was found between CMV IgG levels and total CMV-specific CD4+ T-cell responses (data not shown).

### CMV-specific immune responses and HIV-associated factors

In order to determine whether HIV-associated variables had an impact on CMV-specific immune responses in PLWHIV, we analyzed the association between CMV-specific immune responses and the explanatory variables nadir CD4+ T-cell count, current CD4+ T-cell count, pre-cART viral load and years since diagnosis in a univariate regression model in addition to multivariate regression models adjusted for age and gender. After adjustment, nadir CD4+ T-cell counts were negatively associated with CMV IgG levels (β = −0.49, 95% CI = −0.87;−0.11, p = 0.014), but not with total CMV-pp65- or CMV-gB- specific T-cell responses. In addition, there was a positive association between years since diagnosis of HIV and CMV IgG levels (β = 0.42, 95% CI = 0.04;0.80, p = 0.030), but not with total CMV-specific T-cell responses. Pre-cART viral load and current CD4+ T-cell counts were not associated with CMV-specific immune responses in PLWHIV (data not shown). A total of 58.5% of PLWHIV initiated cART immediate after diagnosis, and we considered the effect of immediate versus delayed initiation of cART on CMV-specific immune responses and found no significant differences between the groups (data not shown).

### No independent associations between CMV IgG levels and immune senescence, immune activation or systemic inflammation in PLWHIV

We further investigated whether PLWHIV with high CMV IgG levels (≥177 U/mL) were characterized by increased markers of T-cell senescence, immune activation or systemic inflammation when compared to PLWHIV with low CMV IgG levels (<177 U/mL). A lower CD4+/CD8+ T-cell ratio was found in PLWHIV with high CMV IgG levels compared to those with low CMV IgG levels (0.6 (0.5;0.9) versus 0.9 (0.7;1.2), p = 0.031), but no other significant differences were found between the groups (Table 1).

Associations between CMV IgG levels and markers of T-cell senescence, immune activation, and systemic inflammation were also investigated in multivariate linear regression models in order to assess independent relationships while adjusting for potential confounders. CD4+/CD8+ T-cell ratio, terminal differentiated versus naïve (TD/N) CD4+ and CD8+ T-cells, senescent CD8+ T-cells, activated CD8+ and CD4+ T-cells, sCD14, hs-CRP, IL-6, and TNF-α were included in individual models as dependent variables and adjusted for age and gender. In addition, separate models were created with addition of a fourth HIV-associated variable in each model (nadir CD4+ T-cell count, viral load before cART, and years since diagnosis). After adjustment for age and gender, a negative association between CMV IgG levels and the CD4+/CD8+ T-cell ratios (β = −1.15, 95% CI = −1.27;−1.04), p = 0.008) was found. However, the association attenuated after adjustment for nadir CD4+ T-cell count (p = 0.064), but otherwise remained stable (Table 2). Additional analysis showed that interaction between nadir CD4+ T-cell count and CMV IgG was not present (p = 0.117). In addition, a weak positive association between CMV IgG levels and TD/N ratios in CD8+ T-cells (β = 1.27, 95% CI 1.01;1.60, p = 0.046) was found. The association attenuated after adjustment with viral load before cART (p = 0.092), but remained stable after adjustment for other variables (Table 2). No association was found between CMV IgG levels and senescent CD8+ T-cells (Table 2). In addition, CMV IgG levels were not associated with markers of inflammation or immune activation (Table 3). Additional adjustment for smoking and body mass index (BMI) did not alter the results (data not shown).

**Table 2:** Multivariate linear regression models for associations between CMV-specific immune responses and markers of immune senescence in PLWHIV.

	**CD4+/CD8+ T-cell ratio**		**TD/N (%CD8)**		**TD/N (%CD4)**		**Senescent CD8+ (%CD8)**	
	**β (95 % CI)**	**p**	**β (95 % CI)**	**p**	**β (95 % CI)**	**p**	**β (95 % CI)**	**p**
**CMV IgG (U/ml)**	**−1.15 (−1.27; −1.04)**	**0.008**	**1.27 (1.01; 1.60)**	**0.046**	**1.15 (−1.25; 1.64)**	**0.449**	**1.05 (−1.07; 1.19)**	**0.358**
Age, years	1.01 **(−**1.01; 1.02)	0.557	1.03 **(−**1.02; 1.07)	0.202	1.02 **(−**1.04; 1.09)	0.477	1.00 **(−**1.02; 1.02)	0.800
Gender, male	1.10 **(−**1.34; 1.64)	0.617	**−**2.01 **(−**4.92; 1.22)	0.125	**−**2.29 **(−**8.99; 0.78)	0.230	**−**1.31 **(−**2.12; 1.24)	0.272
**Separate models for** **CMV IgG** ** – additional inclusion of a HIV-associated variable to each model**								
+ Nadir CD4+, cells/µL	**−**1.09 **(−**1.20; 1.01)	0.064	1.36 (1.07; 1.74)	0.014	1.27 **(−**1.15; 1.84)	0.208	1.07 **(−**1.06; 1.21)	0.317
+ Pre-ART viral load, copies/ml	**−**1.11 **(−**1.22; **−**1.01)	0.044	1.25 (1.04; 1.63)	0.092	1.11 **(−**1.13; 1.61)	0.563	1.05 **(−**1.08; 1.20)	0.445
+ Years since HIV diagnosis	**−**1.14 **(−**1.27; **−**1.03)	0.017	1.36 (1.07; 1.73)	0.015	1.24 **(−**1.18; 1.80)	0.259	1.07 **(−**1.05; 1.22)	0.268
	**CD4+/CD8+ T-cell ratio**		**TD/N (%CD8)**		**TD/N (%CD4)**		**Senescent CD8+ (%CD8)**	
**CMV-pp65 CD8+ T-cells (%)**	**−1.05 (−1.13; 1.03)**	**0.135**	**1.16 (1.00; 1.36)**	**0.051**	**1.03 (−1.24; 1.32)**	**0.797**	**1.11 (1.02; 1.19)**	**0.012**
Age, years	1.00 **(−**1.02; 1.02)	0.870	1.03 **(−**1.02; 1.07)	0.215	1.02 **(−**1.05; 1.10)	0.481	1.00 **(−**1.02; 1.02)	0.914
Gender, male	1.15 **(−**1.32; 1.74)	0.502	**−**2.15 **(−**5.17; 1.12)	0.085	**−**2.24 **(−**9.18; 1.83)	0.254	**−**1.34 **(−**2.11; 1.18)	0.212
**Separate models for total CMVpp65-specific CD8+ T-cell responses – additional inclusion of a HIV-associated variable to each model**								
+ Nadir CD4+, cells/µL	**−**1.05 **(−**1.13; 1.01)	0.108	1.16 **(−**1.02; 1.37)	0.065	1.03 **(−**1.25; 1.32)	0.837	1.11 (1.03; 1.22)	0.020
+ Pre-ART viral load, copies/ml	**−**1.03 **(−**1.11; 1.05)	0.387	1.15 **(−**1.04; 1.37)	0.096	1.04 **(−**1.24; 1.35)	0.765	1.10 (1.02; 1.22)	0.032
+ Years since HIV diagnosis	**−**1.06 **(−**1.15; 1.03)	0.130	1.16 **(−**1.03; 1.37)	0.073	1.05 **(−**1.23; 1.32)	0.706	1.11 (1.03; 1.22)	0.014
	**CD4+/CD8+ T-cell ratio**		**TD/N (%CD8)**		**TD/N (%CD4)**		**Senescent CD8+ (%CD8)**	
**CMV-pp65 CD4+ T-cells (%)**	**−1.09 (−1.18; −1.01)**	**0.034**	**1.27 (1.08; 1.51)**	**0.006**	**1.15 (−1.17; 1.57)**	**0.345**	**1.10 (1.00; 1.19)**	**0.043**
Age (years)	1.00 **(−**1.02; 1.02)	0.928	1.03 (1.00: 1.07)	0.062	1.02 **(−**1.04; 1.09)	0.440	1.00 **(−**1.02; 1.02)	0.892
Gender (male)	1.18 **(−**1.28; 1.77)	0.425	**−**2.42 **(−**4.97; **−**1.18)	0.018	**−**2.85 **(−**10.4; 1.28)	0.110	**−**1.41 **(−**2.17; 1.09)	0.115
**Separate models for** **total CMVpp65-specific CD4+ T-cell responses** ** – additional inclusion of a HIV-associated variable to each model**								
+ Nadir CD4+, cells/µL	**−**1.05 **(−**1.13; 1.02)	0.165	1.29 (1.09; 1.54)	0.005	1.21 **(−**1.07; 1.46)	0.142	1.07 (1.00; 1.14)	0.050
+ Pre-ART viral load, copies/ml	**−**1.06 **(−**1.09; 1.02)	0.159	1.29 (1.07; 1.55)	0.009	1.24 **(−**1.14; 1.68)	0.169	1.10 (1.00; 1.21)	0.040
+ Years since HIV diagnosis	**−**1.09 **(−**1.19; 1.01)	0.065	1.31 (1.11; 1.55)	0.002	1.19 **(−**1.13; 1.63)	0.238	1.10 (1.02; 1.20)	0.022
	**CD4+/CD8+ T-cell ratio**		**TD/N (%CD8)**		**TD/N (%CD4)**		**Senescent CD8+ (%CD8)**	
**CMV-gB CD8+ T-cells (%)**	**−1.08 (−1.16; 1.03)**	**0.027**	**1.25 (1.07; 1.46)**	**0.005**	**1.17 (−1.10; 1.52)**	**0.215**	**1.10 (1.01; 1.20)**	**0.031**
Age, years	1.00 **(−**1.02; 1.02)	0.818	1.04 (1.00; 1.08)	0.081	1.03 **(−**1.04; 1.09)	0.392	1.00 **(−**1.02; 1.02)	0.768
Gender, male	1.20 **(−**1.27; 1.82)	0.393	**−**2.45 **(−**5.86; **−**1.03)	0.043	**−**2.62 **(−**10.6; 1.53)	0.169	**−**1.39 **(−**2.23; 1.15)	0.164
**Separate models for** **total CMVgB-specific CD8+ T-cell responses** ** – additional inclusion of a HIV-associated variable to each model**								
+ Nadir CD4+, cells/µL	**−**1.05 **(−**1.12; 1.05)	0.140	1.28 (1.09; 1.51)	0.003	1.23 **(−**1.07; 1.61)	0.129	1.10 (1.01; 1.21)	0.037
+ Pre-ART viral load, copies/ml	**−**1.04 **(−**1.09; 1.03)	0.031	1.25 (1.06; 1.47)	0.011	1.14 **(−**1.00; 1.47	0.304	1.11 (1.02; 1.19)	0.028
+ Years since HIV diagnosis	**−**1.05 **(−**1.10; 1.02)	0.019	1.26 (1.07; 1.48)	0.006	1.20 **(−**1.09; 1.57)	0.187	1.11 (1.01; 1.21)	0.029
	**CD4+/CD8+ T-cell ratio**		**TD/N (%CD8)**		**TD/N (%CD4)**		**Senescent CD8+ (%CD8)**	
**CMV-gB CD4+ T-cells (%)**	**−1.04 (−1.10; 1.05)**	**0.133**	**1.30 (1.08; 1.56)**	**0.007**	**1.35 (−1.02; 1.66)**	**0.053**	**1.12 (1.02; 1.24)**	**0.024**
Age, years	1.00 **(−**1.02; 1.02)	0.822	1.04 (1.00; 1.08)	0.065	1.03 **(−**1.03; 1.09)	0.363	1.00 **(−**1.02; 1.02)	0.734
Gender, male	1.17 **(−**1.30; 1.78)	0.460	**−**2.44 **(−**5.53; **−**1.08)	0.033	**−**2.76 **(−**10.5; 1.37)	0.133	**−**1.39 **(−**2.21; 1.14)	0.156
**Separate models for** **total CMVgB-specific CD4+ T-cell responses** **– additional inclusion of a HIV-associated variable to each model**								
+ Nadir CD4+, cells/µL	**−**1.03 **(−**1.08; 1.05)	0.215	1.30 (1.08; 1.58)	0.008	1.31 **(−**1.03; 1.72)	0.050	1.12 (1.01; 1.24)	0.027
+ Pre-ART viral load, copies/ml	**−**1.03 **(−**1.09; 1.08)	0.234	1.28 (1.05; 1.56)	0.017	1.22 **(−**1.02; 1.70)	0.233	1.12 (1.00; 1.24)	0.034
+ Years since HIV diagnosis	**−**1.04 **(−**1.10; 1.06)	0.157	1.32 (1.10; 1.59)	0.004	1.23 **(−**1.03; 1.73)	0.218	1.13 (1.03; 1.25)	0.014

Multivariate linear regression analyses with CD4+/CD8+ T-cell ratio, terminally differentiated (TD) (CD45RA+ CD27-CCR7−) versus naïve (N) (CD45RA+ CD27+ CCR7+) T-cells, senescent (CD28-CD57+) T-cells, and chronic activated (CA) (CD38+HLA-DR+) T-cells as dependent variables (all variables are log2-transformed). Results are given as percentage of CD4+ or CD8+ T-cells. Using a backward selection approach on variables with known or potential impact on immune senescense in PLWHIV (age, gender, nadir CD4+ T-cell count, current CD4+ T-cell count, years since diagnosis, pre-ART viral load, smoking and BMI) models were minimized to include only age and gender as covariates. In order to evaluate the effect of the most significant HIV-associated variables, each HIV-associated variable was included in separate models. P-values in bold for p < 0.05. False discovery rate adjusted p-values: CMV IgG versus CD4+/CD8+ p = 0.032, CMV IgG versus TD/N CD8+ p = 0.092, CMV-pp65 CD8+ versus senescent CD8+: p = 0.048, CMV-pp65 CD4+ versus CD4+/CD8+ p = 0.057, CMV-pp65 CD4+ versus TD/N CD8+ p = 0.024, CMV-pp65 CD4+ versus senescent CD8+ p = 0.057, CMV-gB CD8+ versus CD4+/CD8+: p = 0.041, CMV-gB CD8+ versus TD/N CD8+: p = 0.020, CMV-gB CD8+ versus senescent CD8+: *p* = 0.041, CMV-gB CD4+ versus TD/N CD8+: p = 0.028, CMV-gB CD4+ versus senescent CD8+: p = 0.048

Abbreviations: ART, antiretroviral therapy; CMV, cytomegalovirus; IgG, immunoglobulin G; PLWHIV, people living with HIV.

**Table 3:** Multivariate linear regression models for associations between CMV-specific immune responses and markers of immune activation or systemic inflammation in PLWHIV.

Adjusted for age and gender	**CD8** ^**+**^ **CD38** ^**+**^ **HLA-DR** ^**+**^ **(% CD8** ^**+**^ **)**		**CD4** ^**+**^ **CD38** ^**+**^ **HLA-DR** ^**+**^ **(% CD4** ^**+**^ **)**		**sCD14 (pg/mL)**	
	**β (95% CI)**	**p**	**β (95% CI)**	**p**	**β (95% CI)**	**p**
CMV IgG (U/ml)	1.07 (−1.07; 1.22)	0.333	1.12 (−1.01; 1.26)	0.077	−1.01 (−1.25; 1.23)	0.944
Total CMV-pp65 CD8+ (% of CD8+)	1.05 (−1.05; 1.16)	0.297	1.06 (−1.04; 1.16)	0.273	1.00 (−1.17; 1.16)	0.986
Total CMV-pp65 CD4+ (% of CD4+)	**1.13 (1.02; 1.25)**	0.017*	**1.12 (1.00; 1.25)**	0.044*	−1.05 (−1.25; 1.12)	0.534
Total CMV-gB CD8+ (% of CD8+)	1.03 (−1.07; 1.13)	0.568	1.06 (−1.04; 1.16)	0.232	1.01 (−1.16; 1.18)	0.928
Total CMV-gB CD4+ (% of CD4+)	**1.14 (1.01; 1.29)**	0.037*	1.11 (−1.05; 1.25)	0.103	−1.01 (−1.22; 1.20)	0.946
**Adjusted for age and gender**	**IL-6 (pg/ml)**		**TNFα (pg/ml)**		**hsCRP (ug/mL)**	
	**β (95% CI)**	**p**	**β (95% CI)**	**p**	**β (95% CI)**	**p**
CMV IgG (U/ml)	1.06 (−1.07; 1.21)	0.340	1.04 (−1.01; 1.10)	0.120	1.07 (−1.15; 1.33)	0.512
Total CMV-pp65 CD8+ (% of CD8+)	1.00 (−1.10; 1.10)	0.990	1.02 (−1.02; 1.07)	0.248	−1.05 (−1.21; 1.10)	0.483
Total CMV-pp65 CD4+ (% of CD4+)	1.00 (−1.09; 1.13)	0.969	1.03 (−1.01; 1.08)	0.180	−1.19 (−1.39; −1.02)	0.309
Total CMV-gB CD8+ (% of CD8+)	1.00 (−1.11; 1.09)	0.871	1.02 (−1.02; 1.06)	0.276	−1.04 (−1.20; 1.12)	0.629
Total CMV-gB CD4+ (% of CD4+)	−1.02 (−1.14; 1.10)	0.776	1.03 (−1.01; 1.08)	0.164	−1.15 (−1.36; 1.04)	0.120

Multivariate linear regression models with activated CD8+ and CD4+ T-cells (CD38^+^HLA-DR^+^), monocyte activation (sCD14) and systemic inflammation (IL-6, TNFα, hsCRP) as dependent variables and CMV-specific immune responses as independent variables. All variables were log2-transformed. Using a backward selection approach on variables with known or potential impact on immune activation or inflammation in PLWHIV (age, gender, nadir CD4+ T-cell count, current CD4+ T-cell count, years since diagnosis, pre-ART viral load, smoking and BMI) models were minimized to include only age and gender as covariates. P-values in bold for p < 0.05. *False discovery rate adjusted p-value: total CMV-pp65 CD4+ T-cells responses versus CD8+ CD38^+^HLA-DR^+^: p = 0.085^,^ total CMV-gB CD4+ T-cell responses versus CD8+ CD38^+^HLA-DR^+^: p = 0.093^,^ total CMV-pp65 CD4+ T-cells responses versus CD8+ CD38^+^HLA-DR^+^: p = 0.172.

Abbreviations: ART, antiretroviral therapy; CMV, cytomegalovirus, IgG, immunoglobulin G, PLWHIV, people living with HIV.

### CMV-specific T-cell responses were associated with CD8+ T-cell senescence and activation, but not with systemic inflammation

To investigate associations between total CMV-specific T-cell responses and markers of T-cell senescence, immune activation or systemic inflammation we applied the same multivariate linear regression models as when analysing CMV IgG levels. With the exception of CMV-pp65-specific CD8+ T-cells, independent positive associations were found between total CMV-specific T-cell responses and the TD/N ratio in CD8+ T-cells (CMV-pp65 CD4+: p = 0.006, CMV-gB CD8+: p = 0.005, CMV-gB CD4+: p = 0.007, adjusted for age and gender). The associations remained significant after adjustment for nadir CD4+ T-cells, viral load before cART and years since diagnosis in separate multivariate models (Table 2). The total CMV-specific T-cell responses were also associated with higher levels of senescent CD8+ T-cells in multivariate analysis (CMV-pp65 CD8+: p = 0.012, CMV-pp65 CD4+: p = 0.043, CMV-gB CD8+: p = 0.031, CMV-gB CD4+: p = 0.024, adjusted for age and gender), and with the exception of CMV-pp65-specific CD4+ T-cell responses, the associations remained stable after additional adjustment for nadir CD4+ T-cells, viral load before cART and years since diagnosis in separate models (Table 2). When additional adjustment for smoking and BMI was considered in separate models, positive associations remained significant (data not shown). CMV-specific T-cell responses and the TD/N ratios in CD4+ T-cells were not associated, and no independent associations were found between CMV-specific T-cell responses and the CD4+/CD8+ T-cell ratios (Table 2).

In addition, total CMV-specific CD4+ T-cell responses were associated with higher proportions of activated CD8+ T-cells in multivariate models (CMV-pp65: p = 0.017, CMV-gB: p = 0.037, adjusted for age and gender) (Table 3). Additional adjustment for HIV-associated variables did not alter the results. When additional adjustment for smoking and BMI was considered in separate models, the positive associations between CMV-specific CD4+ T-cell responses and activated CD8+ T-cells were still significant (CMV-pp65: p = 0.007, CMV-gB: p = 0.042) (data not shown). A weak association was also found between CMV-pp65-specific CD4+ T-cell responses and chronic activated CD4+ T-cells (p = 0.044), but this association could not be confirmed for CMV-gB-specific CD4+ T-cells (Table 3). No associations were found between CMV-specific CD8+ T-cells and activated T-cells, and none of the CMV-specific T-cell responses were associated with sCD14 (Table 3). Single cytokine T-cell responses (TNF-α, IFN-γ and IL-2) versus CD4+/CD8+ T-cell ratios, TD/N ratios, senescent CD8+ T-cells, and activated T-cells are presented in Supplemental Figure 3.

Finally, we did not find evidence of univariate associations between CMV-specific T-cell responses and systemic inflammation (IL-6, TNF-α, hs-CRP) in treated PLWHIV (Table 3), or in multivariate analysis adjusting for age and gender (Table 3). Additional adjustment for each of the HIV-specific factors or for smoking and BMI in separate models did not alter the results (data not shown).

### Polyfunctional profile of CMV-specific CD8+ and CD4+ T-cells according to the magnitude of the CMV-specific T-cell response and T-cell senescence in PLWHIV

**Figure 3:**
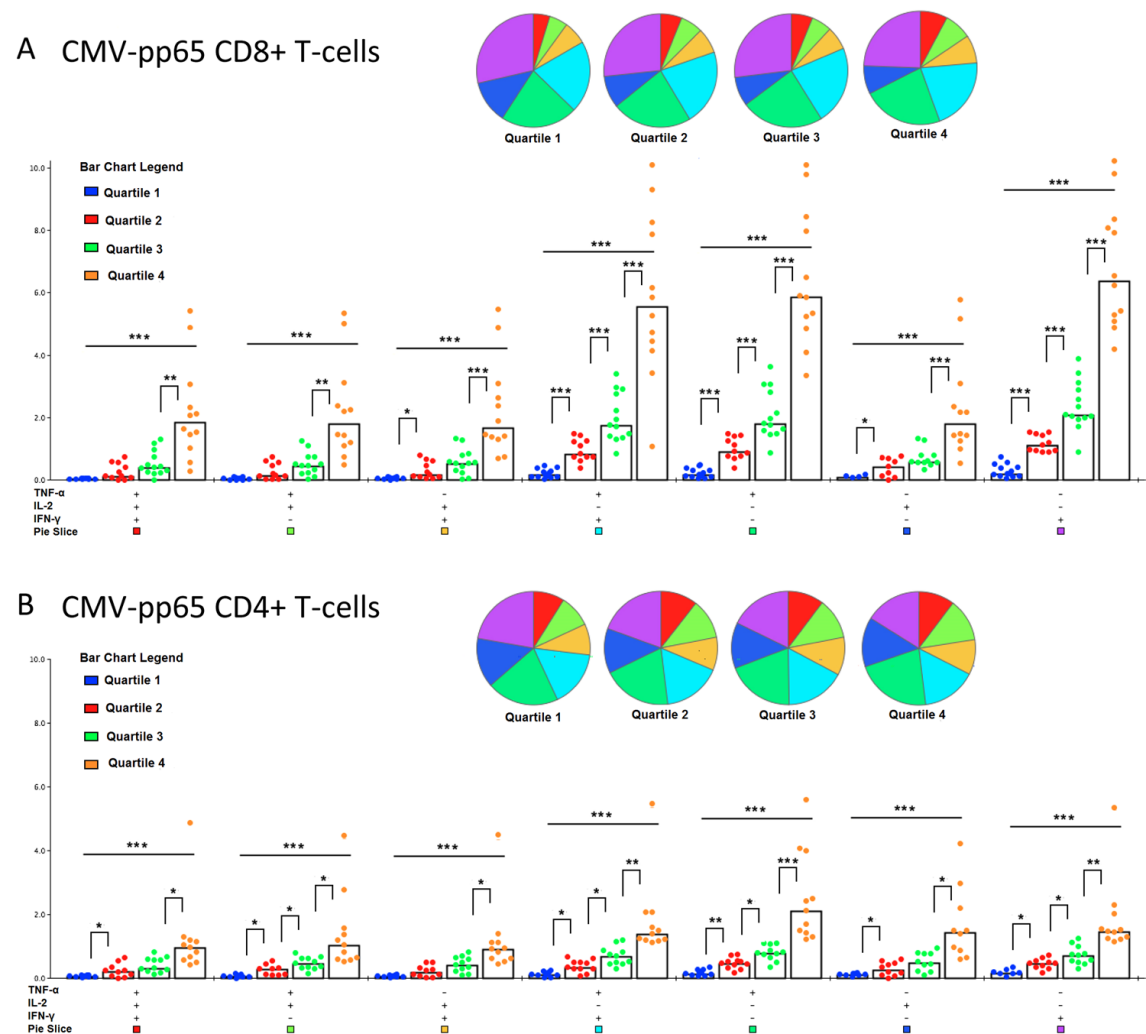
Polyfunctionality profiles of CD8+ and CD4+ CMV-pp65-specific T-cells with increasing total response size. Total CMV-pp65-specific CD8+ and CD4+ T-cell response sizes were separated into quartiles. Pie charts demonstrating the relative contribution of each functional cell subset for each quartile in total CD8+ (**A**) and total CD4+ (**B**) T-cell responses in PLWHIV. Polyfunctionality visualization and analysis was performed using Spice Version 4.2.3. Bar graph illustrates the median frequency of cells in each functional subset for each quartile. Differences between pie charts were tested with permutation tests set at 10.000 repetitions. The difference between quartiles of total CMV-pp65-specific T-cell responses and proportions of polyfunctional T-cell subsets were first tested with the Kruskal-Wallis test and if significant quartile 1 to 2, 2 to 3 and 3 to 4 was compared with the Mann-Whitney U test. *p < 0.01 **p < 0.001. ***p < 0.0001. Bonferroni corrected significance level with 4 end points evaluated; p < 0.013.

The distribution of polyfunctional subsets did not change significantly with increasing total CMV-specific T-cell responses, but for all polyfunctional T-cell subsets, the magnitude of the response increased when the total CMV-pp65-specific CD8+ and CD4+ T-cell response size increased (p ≤ 0.001) (Fig. 3). A similar relationship could not be identified with increasing CMV-gB-specific responses (Supplementary Figure 4).

After adjustment for age, gender and nadir CD4+ T-cell count, CMV-specific CD8+ and CD4+ polyfunctional TNF-α/IFN-γ T-cell responses were associated with an increased TD/N ratio in CD8+ T-cells (CMV-pp65 CD8+: p = 0.029, CMV-pp65 CD4+: p = 0.032, CMV-gB CD8+: p = 0.006, CMV-gB CD4+: p = 0.008) and CMV-specific CD8+ TNF-α/IFN-γ T-cell responses were associated with higher proportions of senescent CD8+ T-cells (CMV-pp65 CD8+: p = 0.032, CMV-gB CD8+: p = 0.032) (Supplementary Table 3). In addition, the CMV-specific IFN-γ/TNF-α CD4+ T-cell responses were associated with chronic activated CD8+ T-cells in univariate analysis, but the association diminished after adjustment with age, gender and nadir CD4+ T-cell count (data not shown). None of the polyfunctional subsets decreased with increasing proportions of CD8+ T-cell senescence markers.

## Discussion

This study provides an analysis of humoral and cellular CMV-specific responses and associations to immune activation, T-cell senescence and systemic inflammation in PLWHIV on stable cART and with low comorbidity. We found, that the magnitudes of CMV-pp65- and CMV-gB-specific T-cell responses were independently associated with several markers of immune senescence, and that CMV-pp65- and CMV-gB-specific CD4+ T-cell responses were associated with activated CD8+ T-cells. However, we did not find evidence of an association between CMV-specific immune responses and systemic inflammation. Furthermore, we observed that T-cell polyfunctionality was maintained despite expansion of CMV-specific T-cells and increasing levels of T-cell senescence markers.

In previous studies, increased CMV-specific CD8+ T-cell responses has been associated with higher proportions of activated CD8+ T-cells (HLA-DR+/CD38+/CD8+) in PLWHIV on cART^15,22^. In addition, a high CMV IgG response has been associated with monocyte activation (sCD14)^20,41^. In contrast to these studies, we did not find associations between CMV IgG responses or CMV-specific CD8+ T-cell responses and immune activation. However, we found an association between increased CMV-specific CD4+ T-cell responses and higher proportions of activated CD8+ T-cells. Notably, previous studies did not investigate CMV-specific CD4+ T-cell responses^15,22^.

In PLWHIV on cART, an association between increased CMV IgG responses and systemic inflammation, defined by increased levels of CRP, CD163, and IL-6 in serum, has previously been reported^20,21,41,42^, and an association between CMV-seropositivity and IL-6 levels has also been found in healthy adults^43^. However, no previous studies have addressed whether this relationship also applies to CMV-specific T-cell responses. In this study, we did not find associations between CMV-specific immune responses and systemic inflammation in PLWHIV on cART, and consequently, the hypothesis that CMV-specific immune responses has a role in driving systemic inflammation during cART was not supported^44^. This could reflect that previous studies were conducted in heterogenous populations, with inclusion of PLWHIV on short-term cART, with low CD4+ T-cell counts, or HCV/HBV coinfection^20,21,41^. In addition, several demographic factors differed, since some of the previous studies were conducted among woman only or in sub-saharan africa^20,21,41^. In the present cohort of PLWHIV all individuals were on long-term stable treatment with cART, had a normalized CD4+ T cell count (mean 592, SD± 256 cells/uL), had suppressed viral replication, no IDU, no co-infection with HCV or HBV or other apparent comorbidities. In studies conducted in heterogeneous populations it may be difficult to distinguish between possible confounders despite statistical adjustment. Despite the strict inclusion criteria’s for this study, we previously found that HIV-positive individuals had increased inflammation and immune activation compared to controls matched on age, gender, BMI and comorbidity^30^. A strength of this study is that confounding factors that may contribute to inflammation and other immune abnormalities are excluded.

In the present study, associations were found between increased magnitude of CMV-specific T-cell responses, increased ratios of terminal differentiated versus naïve CD8+ T-cells, and increased proportions of senescent CD8+ T-cells. These are important findings, since a major concern in the setting of HIV infection is accelerated immune senescence and terminal differentiating of T-cells, which has been associated with increased morbidity and mortality^6^. Several previous studies found that PLWHIV coinfected with CMV had reduced CD4+/CD8+ T-cell ratios and increased levels of CD8+ T-cell senescence markers when compared to those without CMV coinfection^9,10,12,14^, and an association between CMV-specific immune responses and senescent CD8+ T-cells was reported in a small cohort (n = 20) of cART-treated PLWHIV (>50 years) with CD4 nadir <200^23^. However, to our knowledge the present study is the first to confirm this association in a more representative group of PLWHIV and to investigate the impact of HIV-associated factors.

A hallmark of CMV infection in healthy adults are expansion of CD8+ T-cells and maintenance of large proportions of CMV-specific memory CD8+ T-cells^45^. In PLWHIV, proportions of both CD8+ T-cells and CMV-specific CD8+ T-cells are even higher, with the highest levels observed in treated HIV infection^10,46^. CMV infection has been linked to a decreased CD4+/CD8+ T-cell ratio in previous studies^9,10^. In the present study, associations between CMV-specific immune responses and the CD4+/CD8+ T-cell ratio was dependent on the nadir CD4+ T-cell count, suggesting that the nadir CD4+ T-cell count is a crucial factor for the CD4+/CD8+ T-cell ratio as compared to CMV-specific factors. This finding is somewhat expected, since it is well-known that PLWHIV who initiate cART with a low CD4 nadir also have a persistently low CD4/CD8 T-cell ratio^47^. We also found that a low nadir CD4+ T-cell count was associated with increased CMV IgG levels which is consistent with findings from several previous studies^24,48,49^, and indicate a close relationship between initial CD4+ deficiency, CMV reactivation, and long-term humoral CMV-specific immunity. However, not all studies could confirm this association^50,51^, which may be explained by differences in demographic and clinical characteristics between the cohorts investigated.

T-cells were stimulated with peptide-pools from CMV-pp65, CMV-IE1, and CMV-gB in this study. These are well-characterized immunodominant antigens, with CMV-gB being the most frequently recognized by CD4+ T-cells and CMV-pp65 and CMV-IE1 being of the most frequent antigens recognized by CD8+ T-cells in both healthy adults and PLWHIV^52,53^. However, more than 150 CMV antigens are immunogenic for CD8+ and CD4+ T-cells, and it has previously been shown that a total number of 19 specific CMV epitopes are required to predict the total CD8+ and CD4+ T-cell responses to CMV^53^. Thus, when few antigens are investigated only a fraction of the total number of T-cells reacting against CMV will be characterized. Ideally, the entire CMV-specific T-cell response should be characterized, but this requires a very large amount of cells, and selection of immunodominant epitopes may be used as a marker of the general CMV-specific T-cell response. The average total proportion of cells, expressing at least one CMV-specific effector function among all CD8+ or CD4+ T-cells, was 3.5% for CD8+ T-cells and 1.1% for CD4+ T-cells in our study. CMV-specific T-cell responses were comparable with results from a previous study describing CMV-pp65- and CMV-IE1-specific T-cell responses in PLWHIV on cART^46^. In CMV-seropositive HIV-uninfected adults, a comprehensive study previously demonstrated that total CMV-specific T-cell responses comprised approximately 10% of both the CD4+ and CD8+ memory compartments in blood, when all 232 immunogenic peptide mixes were used in a stimulation assay^53^. Thus, the observed pp65-, IE1-, and gB-specific IL2/TNF-α/IFN-γ responses in our study, represents only a small fraction of the total CMV-specific T-cell response, and it is likely that an increase to more than 10% would have been found, if all CMV-specific T-cells were measured.

In this study, the magnitude of the CMV-specific T-cell response was on average a better predictor of immune activation and senescence in PLWHIV than the CMV IgG response. A majority of the previous studies used CMV IgG as a marker of the burden of CMV infection^20,21,24,25,27,28,50^. In future studies, it would be advantageous to include both, since the humoral and cellular CMV-specific immune responses may cover different aspects of CMV immunity in addition to the fact that maintenance of large CMV-specific memory T-cell responses is a specific feature of CMV infection^45^. Recent studies questioned the use of CMV IgG as a surrogate marker of CMV replication and reactivation, since high CMV IgG levels were in fact associated with less CMV replication in semen, and CMV IgG levels were increased after initiation of cART^20,50,54^. Thus, instead of CMV burden, high CMV IgG levels may reflect stronger immune responses to CMV due to other host immune factors or repeated exposures. Interestingly, presence of other herpes virus in semen has been associated with higher CMV IgG levels^50^. Because the presence of other herpes-virus was not investigated, we could not address this question. However, the impact of multiple subclinical viral infections in the setting of treated HIV infection merits further investigation.

In some previous studies, expansion of CMV-specific T-cells and increasing age was associated with decreased polyfunctionality^55,56^, while recent studies found maintained polyfunctionality despite increasing age and phenotypic T-cell alterations in healthy individuals^57–59^. This is also in accordance with findings from the present study. Few previous studies evaluated CMV-specific polyfunctionality in PLWHIV, but recently, maintained polyfunctionality was found in CMV-specific terminally differentiated T-cells from ART-naïve PLWHIV^60^, and in CMV-specific memory CD4+ T-cells from long-term nonprogressors despite inflation of these cells^61^. In accordance with our results, a very recent study showed maintained CMV-specific T-cell polyfunctionality despite phenotypic T-cell alterations in PLWHIV treated with cART^62^. The importance of maintained polyfunctionality is still debated, and an important consideration is, that polyfunctionality may not necessarily be beneficial in PLWHIV, if accompanied by increased immune pathology or tissue damage through high release of cytokines.

### The current study has limitations

The selection of a low-morbid cohort was done to eliminate some important confounders since many previous studies linking CMV with inflammation and immune activation included confounding factors, i.e. HCV infection. It is in this context that the current study takes place, and that the strict selection criteria’s were chosen, but as a consequence results may not be generalizable to the general HIV population or to other demographic locations. Conclusions about causality are not possible because of the cross-sectional design, and the relatively small sample size increases the risk of false negative results. Numerous comparisons were made, which could increase the risk of false positive results. We sought to minimize the risk of false positive results by doing replicate measurements of CMV-specific T-cells. In addition, differences in cytokine responses among CD4+ and CD8+ T-cells were Bonferroni corrected, and when investigating associations between CMV-specific immune responses and markers of immune activation, senescence and systemic inflammation we reported the false discovery rate adjusted p-values for positive associations. Because T-cell function was investigated without MHC multimer staining in our study, a precise quantification of antigen-specific T-cells was not possible, and the impact of HLA alleles on CMV-specific T-cell responses and epitope recognition could not be examined. Another important consideration is the threshold chosen for definition of a positive cytokine response which is of particular importance when investigating polyfunctionality. Many different approaches has been used in recent publications^52,58,61–64^. We chose a threshold defining a positive response as a background-subtracted response above 1/10.000 of CD4+ or CD8+ (>0.01% of CD8+ or CD4+) and at least 40 events. As a sensitivity analysis we applied a threshold of >3 times the background on the CMV-pp65-specific CD4+ and CD8+ T-cell responses. This threshold did not alter the CMV-specific response sizes or the associations to phenotypic T-cell alterations in PLWHIV.

In summary, we found that the magnitude of the T-cell response against the immunodominant CMV epitopes, CMV-pp65 and CMV-gB, but not CMV IgG levels, were associated with a senescent immune phenotype in PLWHIV on stable cART, suggesting that dysfunctional control of CMV or a dysregulated immune response against CMV might contribute to the immunological ageing often reported in PLWHIV. However, we did not find evidence of a relationship between CMV-specific immune responses and systemic inflammation in the present study. In addition, we found maintained CMV-specific polyfunctionality in PLWHIV with expanded CMV-specific T-cell responses or increased T-cell senescence. Further investigations of the complex interactions between CMV, HIV, and the host immune responses are warranted to understand mechanisms underlying aging-related complications during chronic HIV infection.

## Electronic supplementary material

Supplementary information
