# Supplementary material for: Cytomegalovirus-specific T-cells are associated with immune senescence, but not with systemic inflammation, in people living with HIV
Source: Sci Rep. 2018 Feb 28;8:3778. doi: 10.1038/s41598-018-21347-4 (PMC5830877; doi:10.1038/s41598-018-21347-4)
Supplement: Supplementary file 1 — Supplementary information [file 41598_2018_21347_MOESM1_ESM.pdf]

Cytomegalovirus-specific T-cells are associated with immune senescence, but not with systemic inflammation, in people living with HIV

Vibe Ballegaard[1,2], Peter Brændstrup[2,3,4], Karin Kaereby Pedersen[1], Nikolai Kirkby[5], Anette Stryhn[3], Lars P. Ryder[2], Jan Gerstoft[1], Susanne Dam Nielsen[1].

[1]*Viro-immunology Research Unit, Department of Infectious Diseases, Rigshospitalet, University Hospital of Copenhagen, Denmark*

[2]*Department of Clinical Immunology, Rigshospitalet, University Hospital of Copenhagen, Denmark*

[3] *Department of Immunology and Microbiology, University of Copenhagen, Denmark*

[4]*Department of Hematology, Herlev Hospital, Denmark*

[5]*Department of Medical Microbiology, Rigshospitalet, University Hospital of Copenhagen, Denmark.*

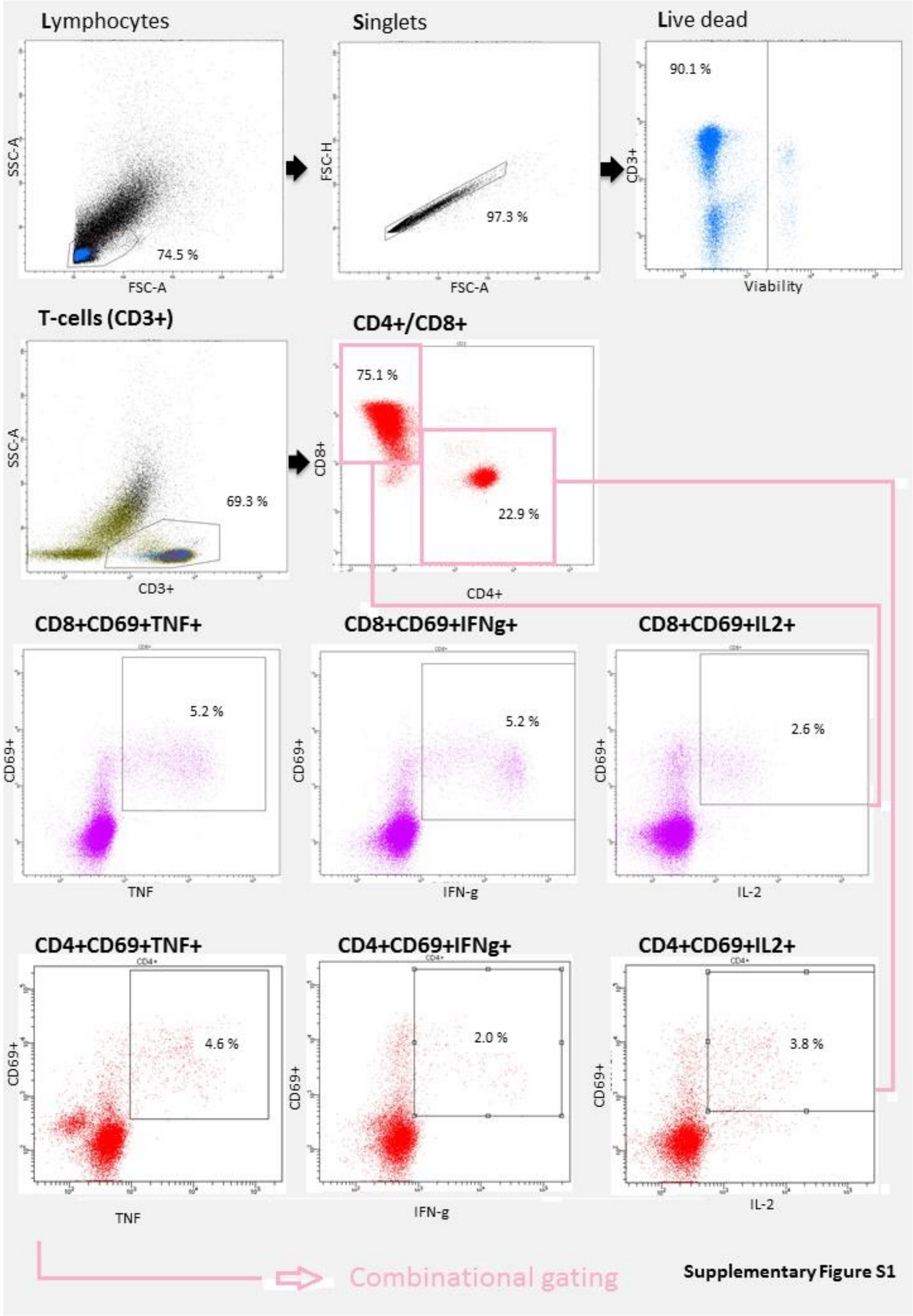

Supplementary Figure 1: Gating strategy for intracellular cytokine staining. A lymphocyte gate based on FSC/SSC, a singlet gate, and a live/dead cell gate were applied before gating on CD3+CD4+ and CD3+CD8+ cells. Further, for each T-cell subset, CD69+ populations were gated from CD69+ histograms for CD4+ and CD8+ populations, and expression of IFN-γ, TNF-α and IL-2 was then determined from the CD4+ and CD8+ populations. To obtain co-expression patterns a combinational gating strategy was applied to obtain all functional subsets.

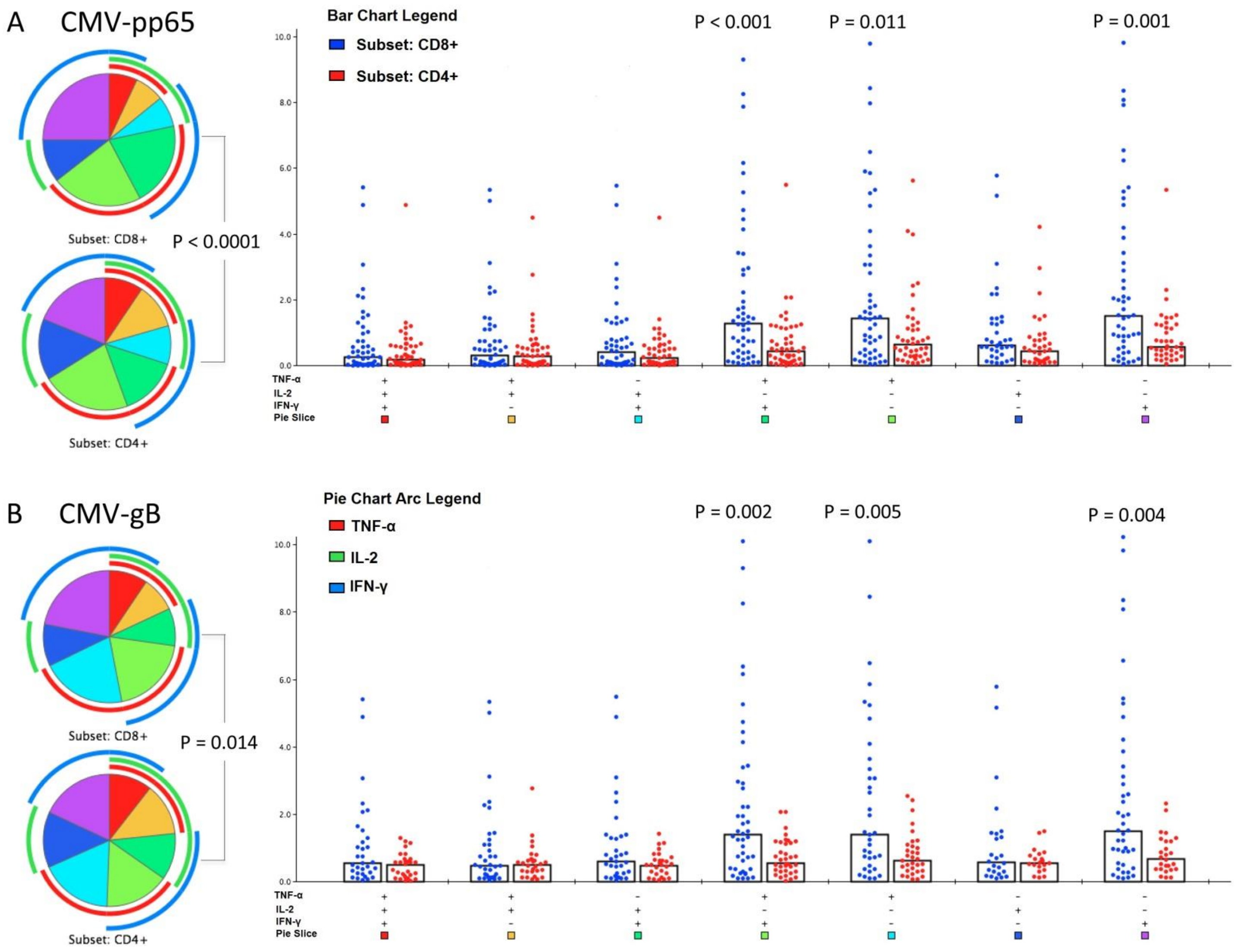

Supplementary Figure 2: Polyfunctionality profiles of CD8+ and CD4+ CMV-specific T-cells in PLWHIV. The profile of CMV-pp65-, CMV-gB-, and CMV-IE1- specific T-cell function was assessed with measurement of IFN-γ, TNF-α, and IL-2 in PLWHIV. Polyfunctionality analysis of CMV-IE1 T-cell function was not performed due to a low response frequency. Polyfunctionality visualization and analysis was performed using Spice Version 4.2.3. Pie charts demonstrate the relative contributions of each functional cell subset among total CD8+ and CD4+ T-cell responses. Bar graphs illustrate the median frequency of cells in each functional subset among CD8+ and CD4+ T-cells in **(A)** CMV-pp65-specific T-cells and **(B)** CMV-gB-specific T-cells. Differences between pie charts were tested with permutation tests set at 10.000 repetitions (Roederer et al. *Cytometry 2011*). The difference between CD8+ and CD4+ cells was tested with Wilcoxon paired signed rank test. Significance level was set at $P<0.05$.

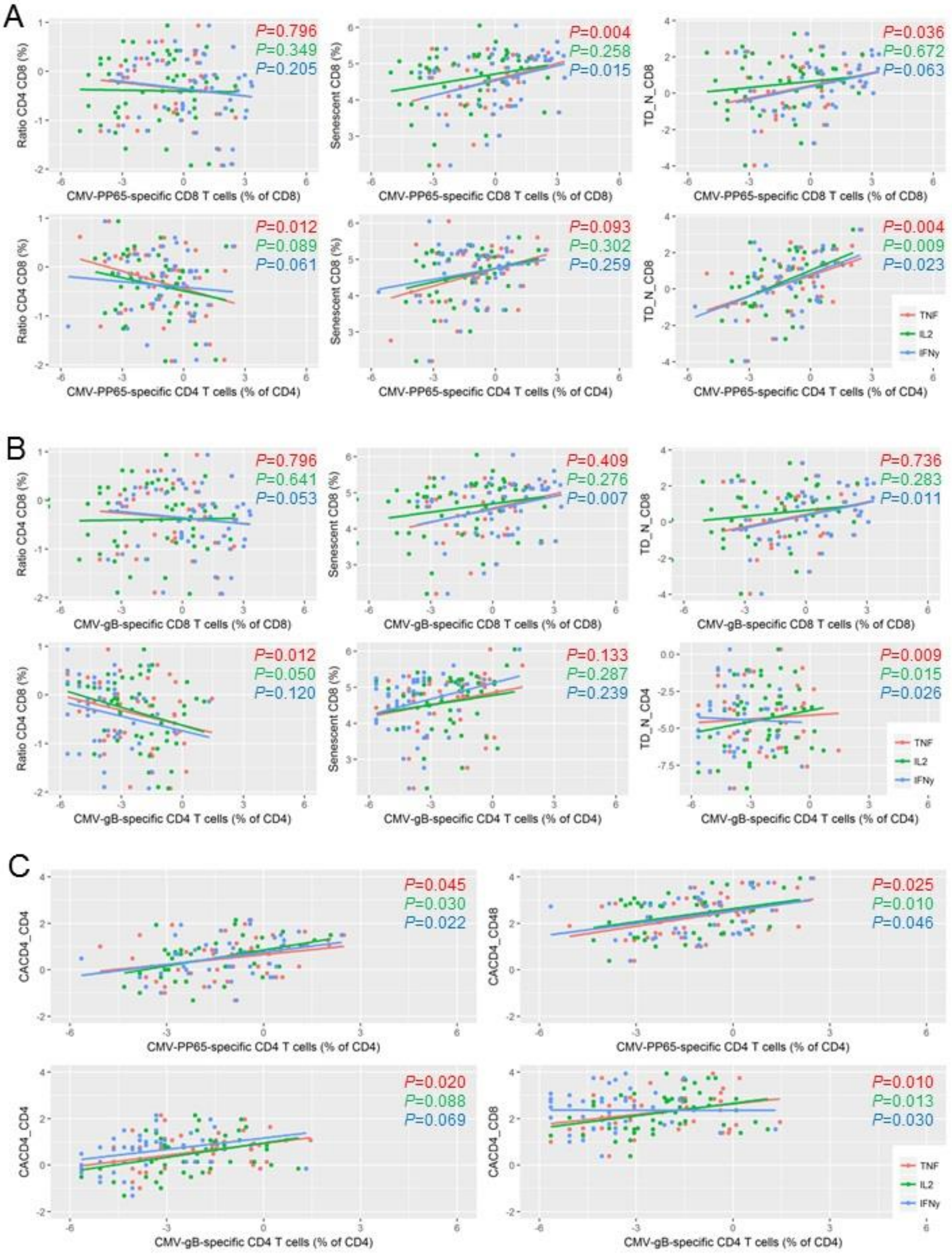

Supplementary Figure 3: CMV-specific CD4+ and CD8+ T-cell responses at a single cytokine level (TNF-α, IFN-γ or IL-2) and associations to CD4+/CD8+ T-cell ratios, senescent CD8+ T-cells (% of CD8+ T-cells) and ratio of terminally differentiated versus naïve (TD/N) T-cells after **(A)** CMV-pp65 stimulation and **(B)** CMV-gB stimulation**.** In **(C)**, associations between CMV-pp65- and CMV-gB-specific CD4+ T-cell responses and activated CD4+ and CD8+ T-cells is showed. All data are log-transformed. A univariate general linear model was used for analyzing associations between single cytokine responses and T-cell senescence or chronic immune activation. Cytokine responses and *P*-values are marked in red for TNF-α, in green for IL-2, and in blue for IFN-γ.

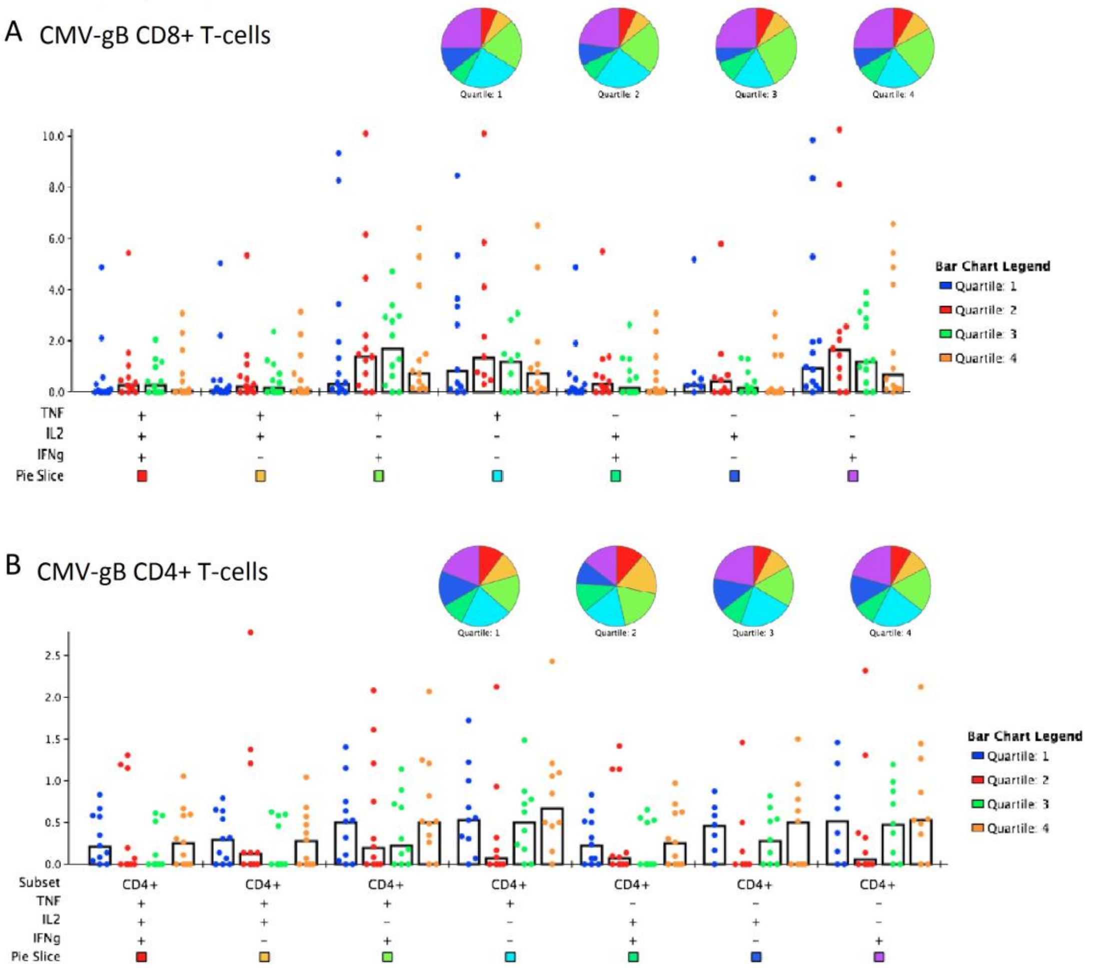

Supplementary Figure 4: Polyfunctionality profiles of CD8+ and CD4+ CMV-gB-specific T-cells with increasing total response size. Total CMV-gB-specific CD8+ and CD4+ T-cell response sizes were separated into quartiles. Pie charts demonstrating the relative contribution of each functional cell subset for each quartile in total CD8+ **(A)** and total CD4+ **(B)** T-cell responses in PLWHIV. Polyfunctionality visualization and analysis was performed using Spice Version 4.2.3. Bar graph illustrates the median frequency of cells in each functional subset for each quartile. Differences between pie charts were tested with permutation tests set at 10.000 repetitions.

Supplementary table 1. Associations between polyfunctional CMV-specific T-cell responses and markers of immune senescence in PLWHIV

**Explanatory variable**	**TD/N (% of CD8)**			**Senescent CD8+ (% CD8)**		
**CMV-pp65 CD8+ (% of CD8+ T-cells)**	***B (95% CI)***	***P***	***M (P)***	***B (95% CI)***	***P***	***M (P)***
IFN-γ, TNF-α, IL-2	1.08 (-1.05-1.23)	0.200	0.199	1.05 (-1.00-1.12)	0.088	0.123
IFN-γ,TNF-α	1.16 (1.01-1.34)	0.046	0.029	1.10 (1.02-1.18)	0.014	0.011
IFN-γ, IL-2	1.15 (-1.07-1.40)	0.168	0.292	1.05 (-1.02-1.13)	0.146	0.128
TNF-α, IL-2	1.08 (-1.06-1.23)	0.292	0.366	1.07 (-1.03-1.17)	0.155	0.243
**Explanatory variable**	**TD/N (% of CD8)**			**Senescent CD8+ (% CD8)**		
**CMV-pp65 CD4+ (% of CD4+ T-cells)**	***β (95% CI)***	***p***	***M (p)***	***β(95% CI)***	***P***	***M (p)***
IFN-γ, TNF-α, IL-2	1.17 (1.00-1.37)	0.057	0.014	1.03 (0.95-1.12)	0.443	0.357
IFN-γ,TNF-α	1.20 (1.02-1.42)	0.032	0.011	1.08 (1.00-1.17)	0.094	0.082
IFN-γ, IL-2	1.14 (-1.02-1.32)	0.095	0.041	1.04 (-1.03-1.12)	0.352	0.211
TNF-α, IL-2	1.23 (1.04-1.47)	0.018	0.008	1.03 (-1.06-1.11)	0.517	0.488
**Explanatory variable**	**TD/N (% of CD8)**			**Senescent CD8+ (% CD8)**		
**CMV-gB CD8+ (% of CD8+ T-cells)**	***β (95% CI)***	***p***	***M (p)***	***β (95% CI)***	***p***	***M (p)***
IFN-γ, TNF-α, IL-2	-1.08 (-1.36-1.17)	0.501	0.209	-1.01 (-1.11-1.10)	0.966	0.816
IFN-γ,TNF-α	1.22 (1.04-1.43)	0.016	0.006	1.10 (1.02-1.20)	0.019	0.032
IFN-γ, IL-2	1.00 (-1.02-1.23)	0.871	0.958	-1.01 (-1.12-1.09)	0.764	0.844
TNF-α, IL-2	1.00 (-1.03-1.20)	0.799	0.925	-1.01 (-1.11-1.09)	0.839	0.923
**Explanatory variable**	**TD/N (% of CD8)**			**Senescent CD8+ (% CD8)**		
**CMV-gB CD4+ (% of CD4+ T-cells)**	***B (95% CI)***	***P***	***M (P)***	***B (95% CI)***	***P***	***M (P)***
IFN-γ, TNF-α, IL-2	1.29 (1.04-1.59)	0.009	0.007	1.08 (-1.02-1.19)	0.124	0.138
IFN-γ,TNF-α	1.24 (1.01-1.53)	0.013	0.008	1.06 (-1.04-1.17)	0.288	0.331
IFN-γ, IL-2	1.20 (1.01-1.58)	0.043	0.025	1.09 (-1.04-1.23)	0.157	0.186
TNF-α, IL-2	1.22 (1.00-1.58)	0.031	0.019	1.11 (-1.02-1.25)	0.088	0.084

Univariate regression models with terminally differentiated (TD) (CD45RA+CD27-CCR7-) versus naïve (N) (CD45RA+CD27+CCR7+) T-cells and senescent (CD28-CD57+) CD8+ T-cells as dependent variables. Results are given as percentage of CD4+ or CD8+ T-cells. Age, gender, and nadir CD4+ T-cells were included in a multivariate model (M). All variables are log2-transformed.
